# Dystroglycan Depletion Impairs Actin-Dependent Functions of Differentiated Kasumi-1 Cells

**DOI:** 10.1371/journal.pone.0144078

**Published:** 2015-12-02

**Authors:** Marco Antonio Escárcega-Tame, Ivette Martínez-Vieyra, Lea Alonso-Rangel, Bulmaro Cisneros, Steve J. Winder, Doris Cerecedo

**Affiliations:** 1 Laboratorio de Hematobiología, Escuela Nacional de Medicina y Homeopatía (ENMH), Instituto Politécnico Nacional (IPN), Mexico City, Mexico; 2 Departamento de Genética y Biología Molecular, Centro de Investigación y de Estudios Avanzados del IPN (Cinvestav-IPN), Mexico City, Mexico; 3 Department of Biomedical Science, University of Sheffield, Sheffield, United Kingdom; INSERM U894, FRANCE

## Abstract

**Background:**

Dystroglycan has recently been characterised in blood tissue cells, as part of the dystrophin glycoprotein complex involved in the differentiation process of neutrophils.

**Purpose:**

In the present study we have investigated the role of dystroglycan in the human promyelocytic leukemic cell line Kasumi-1 differentiated to macrophage-like cells.

**Methods:**

We characterised the pattern expression and subcellular distribution of dystroglycans in non-differentiated and differentiated Kasumi-1 cells.

**Results:**

Our results demonstrated by WB and flow cytometer assays that during the differentiation process to macrophages, dystroglycans were down-regulated; these results were confirmed with qRT-PCR assays. Additionally, depletion of dystroglycan by RNAi resulted in altered morphology and reduced properties of differentiated Kasumi-1 cells, including morphology, migration and phagocytic activities although secretion of IL-1β and expression of markers of differentiation are not altered.

**Conclusion:**

Our findings strongly implicate dystroglycan as a key membrane adhesion protein involved in actin-based structures during the differentiation process in Kasumi-1 cells.

## Introduction

Hematopoietic stem cells (HSC) are multipotent cells that have the potential to differentiate into all different blood cell types, whilst retaining HSC potential through numerous cell divisions, by a process named haematopoiesis. Intrinsic and extrinsic cues regulate the behaviour of HSC and protect them from exhaustion [1,2]. A number of extracellular matrix (ECM) and cell adhesion proteins have been implicated as having effects on regeneration, differentiation, attachment and migration, and are important factors in the development and progression of many types of cancer [3].

Dystroglycan is an important adhesion molecule and signalling scaffold described in several cell types and tissues and is involved in several disease processes [4]. Dystroglycan (Dg) comprises two glycoproteins that are post-translationally cleaved from a single gene. The extracellular peripheral membrane subunit α-dystroglycan (α-Dg) undergoes extensive glycosylations by including mucin type O-glycosylation, O-mannosylation, and N-glycosylation. A central mucin-like central region of α-Dg is particularly important for interactions between α-Dg and extracellular matrix proteins such as agrin, perlecan and laminin [5], whilst its C-terminal domain interacts noncovalently with the N-terminal extracellular domain of the β-subunit. β-Dg crosses the membrane, and its cytosolic domain is anchored to actin through the interaction with dystrophin, utrophin and other cytoskeletal linker proteins [4,6].

The Kasumi-1 cell line was derived from the peripheral blood of a 7-year-old Japanese boy diagnosed as Acute Myeloid Leukaemia (AML) FAB M2 in relapse after bone marrow transplantation and expresses a 8:21 chromosome translocation [7]. The Kasumi-1 cells can differentiate into macrophage-like cells when treated with phorbol ester, 12-0-tetradecanoylphorbol-13-acetate (TPA) [8].

Recently, we described the role of Dg in HL-60 cells with an active participation in the chemotaxis, phagocytosis and differentiation process to human neutrophils [9]. In the present work we describe the pattern expression and subcellular distribution of dystroglycans in differentiated and non-differentiated Kasumi-1 cells. Our results suggest a dynamic traffic in the cellular compartments and differential expression of dystroglycan species, characteristic of cell linage and its physiological conditions. Additionally we investigated the key role Dg plays in actin-based structure assembly and differentiation process in macrophage-like cells.

## Materials and Methods

### Kasumi-1 Cell culture and differentiation

Kasumi-1 cells were cultured in RPMI-1640 medium supplemented with 10% fetal bovine serum, 400 mM L-glutamine, 50 μM gentamycin, 25 mM HEPES, 2 g/L sodium bicarbonate, 1 mM sodium pyruvate in a humid atmosphere of 5% CO_2_ at 37°C. For differentiation into a macrophage like cells, Kasumi-1 cells were differentiated (dKasumi-1) with 10−^7^ M 12-0-tetradecanoylphorbol-13-acetate (TPA) for 7 days [7]. Cell viability was assessed by exclusion of 0.2% trypan blue and was routinely >90% before and after differentiation.

### Treatment of Kasumi-1 cells with cytoskeleton inhibitor

For morphological analysis, differentiated and non-differentiated Kasumi-1 cells (1 x 10^5^) were incubated with the same volume of the drug in order to obtain final concentrations of 10 μmol of Cytochalasin D in DMSO [10] for 60 min at room temperature. Equivalent final amounts of DMSO were added to control cultures. For differentiation markers, differentiated Kasumi-1 cells (1 x 10^5^) were simultaneously incubated with 10 μmol of Cytochalasin D in DMSO or DMSO and 10−^7^ M 12-0-tetradecanoylphorbol-13-acetate (TPA) for 7 days.

### Immunofluorescence staining

Antibodies used: α-dystroglycan clone VIA4-1 monoclonal antibody Cat. no. 05–298, α-dystroglycan clone IIH6C4, α-dystroglycan clone 6C1 and GAPDH MAB374 were purchased from Millipore (Billerica, MA, USA), β-dystroglycan Cat. no. sc-30405 monoclonal antibody Cat. no. sc-21012, were purchased from Santa Cruz Biotechnology, Inc. (Santa Cruz, CA, USA), β-dystroglycan PY892 Cat. no. 617102 was purchased from Biolegend, (San Diego, CA, USA).

Kasumi-1 cells were adhered to poly-D-lysine-coated coverslips and after 60 minutes permeabilised and fixed with a mixture of 2% p-formaldehyde, 0.04% NP40 in the cytoskeleton stabilizing solution PHEM and triton 0.2%. All the immunofluorescence procedures have been described before [9].

### Morphological analysis

Filopodia size and number were detected using the software FiloDetect from images taken from fluorescence microscopy [11].

### Western blotting analysis

Kasumi-1 cells were resuspended and lysed with a 2X lysis buffer (0.5% NP-40, 2 mM Na_3_VO_4_, PMSF) containing a protease inhibitor cocktail. Homogenates were sonicated 3 times for 15 seconds. The protein concentrations were determined by the BCA method. The protein extract was mixed with loading buffer (Tris–HCl sodium dodecyl sulfate, β-mercaptoethanol, glycerol, bromophenol blue) and boiled for 5 minutes. Whole cell lysates were separated as described in [9].

### Plasmids and transfection

shRNA constructs targeting dystroglycan or control shRNA were generated using the RNAi- Ready pSiren-RetroQ system (Clontech Laboratories Inc. Palo Alto, CA, USA) and have been described previously [9]. Kasumi-1 cells (2 × 10^6^/ ml) were transfected with 2μg DNA mixed with 2 μg pEGFP-N1 to mark the shRNA transfected cell population (Clontech Laboratories Inc. Palo Alto, CA, USA) on the Nucleofector II machine (Amaxa), according to the manufacturer's recommendation (Lonza Walkerrsville Inc. Walkersville, MD). Twenty-four hours after transfection cells were selected for cell sorting (Moflo XDP, Beckman Coulter, Brea, CA, USA). For functional assays, selected cells were differentiated for 7 d.

### Migration assay

The effect of dystroglycan depletion on Kasumi-1 differentiated cells on migration was evaluated using a 12-well Transwell chamber (Corning, Costar Corp Cambridge, MA, USA). Kasumi-1 differentiated cells were added to the upper chambers and allowed to migrate for 120 min at 37°C in 5% CO_2_ towards the lower chamber containing 10^−7^ LPS. Migrated cells from lower chamber were centrifuged at 1200 rpm for 5 min, collected at the bottom of the lower chambers and quantified; the result was expressed as a percentage of cells added to the top of the plate.

### Quantitative real-time PCR

Total RNA was extracted from Kasumi-1 using Trizol Reagent (Life Technologies, Waltham, MA, USA) according to the manufacturer’s instruction. 100 ng of total RNA was subjected to real-time PCR performed in triplicate using KAPA SYBR FAST (one step qRT-PCR kit, Kapa Biosystems, Wilmington, MA, USA) in a final volume of 20 μl. Reactions without a template or without the reverse transcriptase were used as negative controls as described in [9].

### Analysis of cell-surface antigens by immunofluorescence flow cytometry

1 X 10^5^ cells/ml of transfected and control Kasumi-1 cells differentiated for 7 days were incubated with monoclonal antibody for 30 min at 4°C, washed twice with phosphate-buffered saline, and suspended for 30 min at 4°C in 100 μl of Alexa 488-conjugated goat anti-rabbit IgG (Molecular probes Kallestad Laboratories, Inc., Austin, TX) as previously described [9]. Results were shown as percentages of positive cells for each antigen.

### Phagocytosis assays

1 X 10^5^ cells/ml of transfected and control Kasumi-1 cells differentiated for 7 days were allowed to interact at 37°C with Phalloidin-TRITC-labeled *Candida glabrata*, as described in [9].

### Enzyme-linked immunosorbent assays (ELISAs) for IL-1β

1 X 10^5^ cells/ml of transfected and control Kasumi-1 cells differentiated for 7 days in 12-well plates (Corning, Costar Corp Cambridge, MA, USA). The cultures were prepared in duplicate and stimulated either with *E*. *coli* LPS at 1 μg/ml, after 24h of incubation the supernatants were collected and assayed for secreted IL-1β levels. Cytokine levels were measured according to the manufacturer’s instructions by using the human IL-1β (Enzo Life Sciences Inc; Farmingdale, NY, USA).

### Laminin Overlay Assay

Differentiated and non-differentiated Kasumi-1 cells extracts enriched by WGA affinity chromatography, were transferred to a nitrocellulose membrane. The membrane was blocked with low-fat milk/Laminin-binding buffer (LBB) (19 mM triethanolamine-HCl, 140 mM NaCl, 1mM CaCl_2_, 1 mM MgCl_2_, pH7.4) at room temperature and was washed with 3% BSA/LBB. The blot was incubated with 10 nM laminin (L2020, Sigma-Aldrich, St. Louis, MO, USA) in 3% BSA/LBB at 4°C for >12 h. After washing with 5% skim milk/LBB, the blot was incubated with anti-laminin (SC-5582, Sta. Cruz, CA, USA) and IIH6C4 antibodies overnight. After washing with 5% skim milk/LBB, the blot was incubated with the appropriate HRP-conjugated secondary antibody at room temperature for 1 h. The blot was developed by enhanced chemiluminiscence.

### Cell fractionation

To obtain nuclear extracts, differentiated and non-differentiated cells were washed twice with 2 ml of ice-cold PBS and collected by centrifugation at 1800 rpm for 15 min at 4°C. The cell pellet was resuspended in buffer A [10mM NaCl, 10 mM Tris–HCl pH 8.0, 3 mM MgCl_2_, 0.5% NP40] supplemented with complete 1X (Roche Applied Science, Indianapolis, IN, USA) and phosphatase inhibitors [2 mM Na_3_VO_4_ and 25 mM NaF] and incubated for 1 h on ice. Then, buffer B [(v/v) buffer A and 0.6M sucrose] was added, the homogenate was incubated for 15 min on ice and transferred to a glass Dounce homogenizer, stroked 40 times with B pestle, and centrifuged at 6000 rpm for 10 min at 4°C. The supernatant was saved as cytoplasmic extract. The nuclear pellet was once washed with (v/v) of buffer A and buffer B at 6000 rpm/5 min and three times at 6000rpm/5 min with buffer C [(v/v) buffer A without NP40 and buffer B]. Nuclei were recovered with 2X lysis buffer (20 mM Tris–HCl pH 8.0, 1 mM MgCl_2,_ complete 1X, 2 mM Na_3_VO_4_ and 25 mM NaF), 1 volume of 4% SDS and 3 volumes of 1X lysis buffer incubated for 1 h at 4°C, and pre-cleared at 13,000 rpm for 2 min at 4°C. The supernatant was saved as nuclear extract.

### Statistical analysis

Statistical analysis was carried out with GraphPad Prism for Windows ver5 software (GraphPad Software, Inc., La Jolla, CA, USA). Unpaired T-test and One-way Analysis of variance (ANOVA) were utilized for data analysis.

## Results

### Dystroglycan is present in Kasumi-1 cells

To determine the pattern expression of dystroglycan (Dg) in differentiated (dKasumi-1 cells) and non-differentiated Kasumi-1 cells (ndKasumi-1), we performed western blot (WB) assays using antibodies directed to laminin binding glycans present in α-Dg, antibodies raised against β-Dg and β-DgPY892. The molecular mass of α-Dg varies from 120 to 200 kDa, depending on the extent of its glycosylation and tissue source [12].

**Fig 1:**
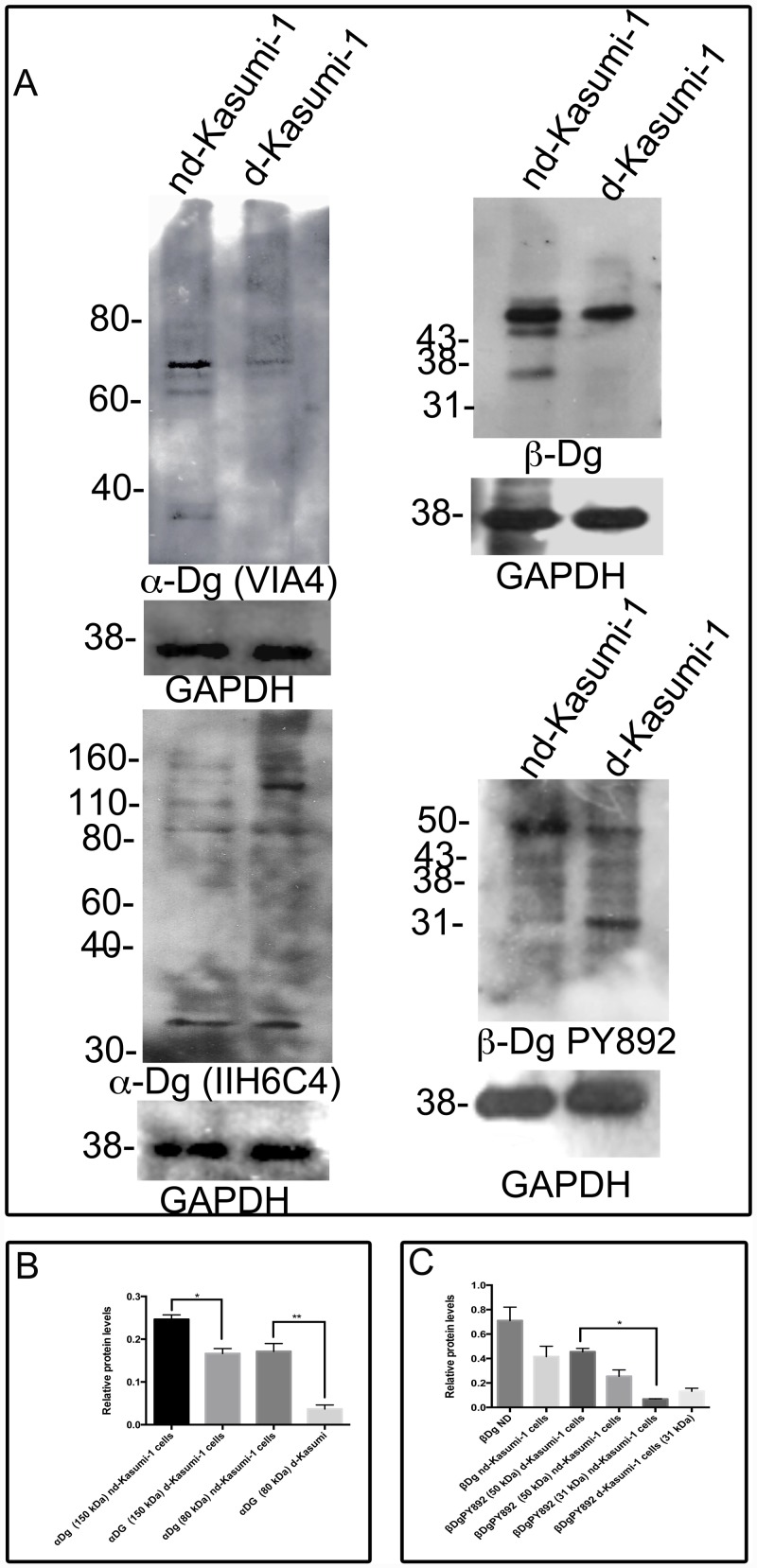
Pattern expression of dystroglycans in Kasumi-1 cells. Total lysates of differentiated (d-Kasumi-1 cells) and non-differentiated Kasumi-1 cells (nd-Kasumi-1) were analysed by western blot utilizing antibodies against C-terminal of α-Dg, β-Dg and β-DgPY892. Quantitative analysis using GAPDH as loading control is shown. Values shown are mean ±SD from three independent experiments (n = 3), respectively. * P<0.05.

We detected with VIA4 antibody a prominent protein band of 70 kDa in nd-Kasumi-1 cells compared with dKasumi-1. Kasumi-1 cells for these assays were cultured in the presence of decanoyl-RVKR-CMK, a furin inhibitor. Other bands of 62 kDa and 35 kDa were observed only in ndKasumi-1 cells extracts; bands of higher relative mass were not apparent under this treatment and with the antibody used. However nd-Kasumi-1 and d-kasumi-1 cell lysates enriched with WGA showed the presence of the full-length α-Dg of 150 kDa revealed with IIH6C4 antibody. Densitometric analysis of α-Dg species of 150 kDa and 80 kDa showed that both were more expressed in Kasumi-1 non-differentiated cells (Fig 1B).

β-Dg was observed with bands of 43 kDa, 38 kDa and a faint band of 31 kDa, these last two bands were more evident for ndKasumi-1 cells. β-Dg phosphorylated on tyrosine 892 (β-DgPY892) was revealed by the presence of 50 kDa bands in both samples but was more apparent in nd-Kasumi-1, in contrast a band corresponding to 31 kDa was more evident in d-Kasumi-1 cells. The presence of bands corresponding to 43 kDa and 35 kDa were equivalent in Kasumi-1 differentiated and Kasumi-1 non-differentiated cells (Fig 1C).

Relative protein levels showed a statistical significance of α-Dg 150 kDa band with mean values of 0.2465 and 0.1664 for non-differentiated and differentiated Kasumi-1 cells respectively; and 80 kDa band with values of 0.1709 and 0.0365 for non-differentiated and differentiated Kasumi-1 cells respectively. Relative protein mean values for β-DgPY892 (50 kDa) corresponded to 0.4555 and (31 kDa) 0.0687 for non-differentiated Kasumi-1; while for differentiated Kasumi-1 cells, relative values expression for 50 k Da and 31 k Da species were 0.2542 and 0.1322 respectively (Fig 1A). Considering the relative protein levels we compared the proportion of β-DgPY892 of 31 kDa in relation to the sum of the different reactive bands detected with the respective antibody and the mean value registered to the nd-Kasumi-1 cells represent the half of the mean value observed for d-Kasumi-1 cells (S1 Fig).

**Fig 2:**
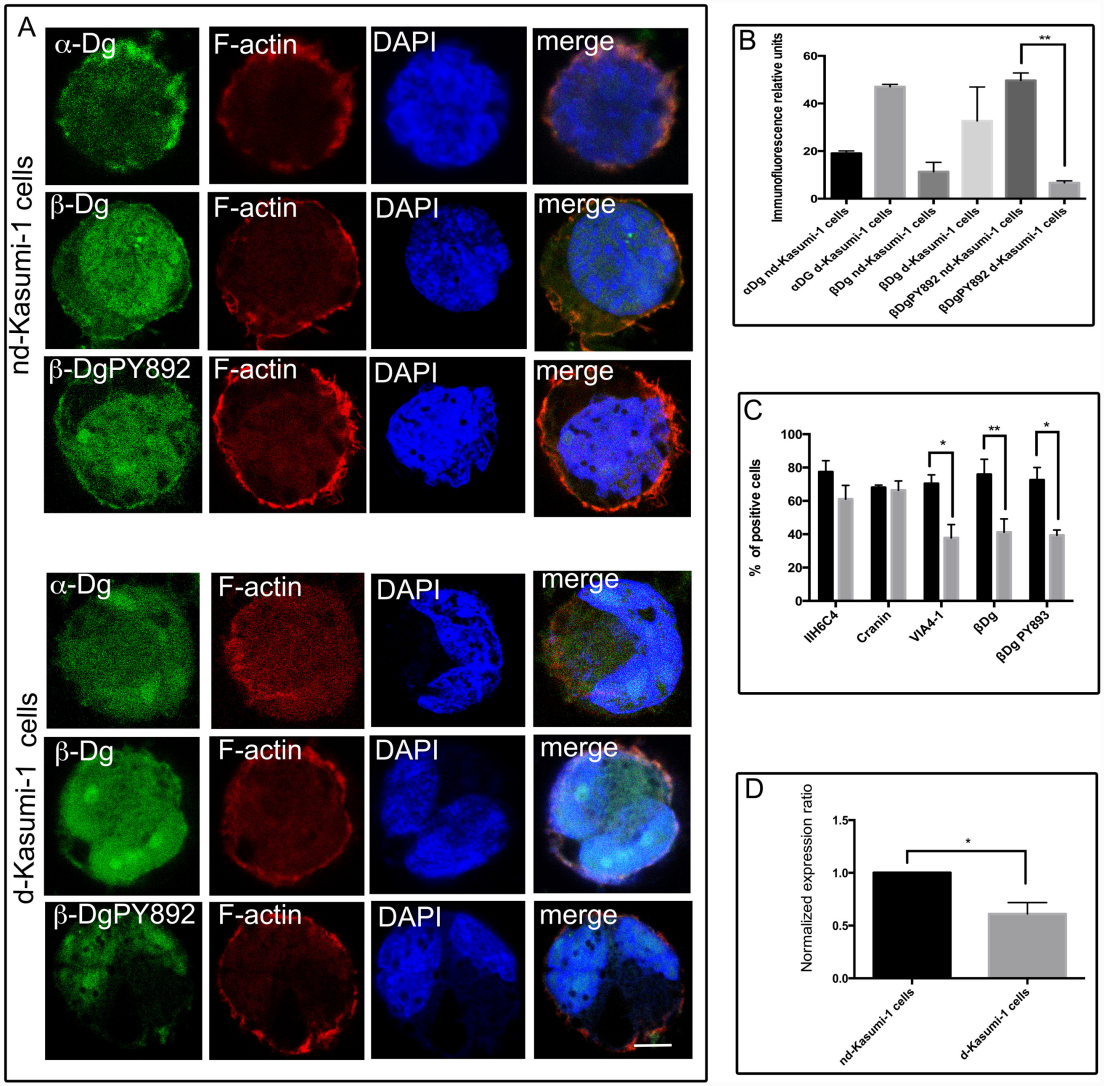
Subcellular distribution of dystroglycans in Kasumi-1 cells. (A) Non-differentiated (nd-Kasumi-1 cells) and differentiated Kasumi-1 cells (d-Kasumi-1 cells) settled on glass cover slips were processed for double immunofluorescence and analysed by confocal microscopy. Bar = 5μm. (B) Respective immunofluorescence intensities were quantified. Values shown are mean ±SD from three independent experiments (n = 3), respectively. ***P<0.005. (C) FACS analysis of nd-Kasumi-1 (black boxes) cells showed reduced expression of laminin-binding glycans on α-Dg detected by IIH6C4 and VIA4, β-Dg and β-DgPY892 compared with d-Kasumi-1 cells (grey boxes). (D) Messenger RNA expression of dystroglycan was examined by quantitative reverse transcription PCR in control and differentiated Kasumi-1 cells. Values shown are mean ± SD from three independent experiments (n = 3), respectively. *P<0.05. (nd-Kasumi-1) non-differentiated cells; (d-Kasumi-1) differentiated cells.

To evaluate the subcellular distribution of Dg subunits, double immunofluorescence (IF) staining and confocal microscopy analysis on differentiated Kasumi-1 (d-Kasumi-1 cells) and non-differentiated Kasumi-1 cells (nd-Kasumi-1 cells), were performed utilizing antibodies raised against α-Dg, β-Dg and β-DgPY892 revealed with Alexa-Fluor-488 secondary antibody. Actin filaments were identified with Tetramethyl rhodamine iso-thiocyanate (TRITC)-phalloidin. In nd-Kasumi-1 cells, α-Dg subunit displayed a punctuated pattern distributed mainly at the plasma membrane where it co-localized with actin filaments. This distribution was very similar to that observed in differentiated cells, although α-Dg was also observed in the cytoplasm. In both d-Kasumi-1 and nd-Kasumi-1 cells, β-Dg was observed with a discrete patched pattern at the cytoplasm, but in differentiated cells the label was strongly observed in the nucleus. Apparently most of the β-Dg corresponded to its phosphorylated form, which was also increased and mainly located in the nucleus. The relative fluorescence intensities showed values statistically significant for α-Dg and β-DgPY892 in Kasumi non-differentiated compared to Kasumi-1 differentiated cells (Fig 2B).

To evaluate if there were variations of expression in laminin-binding glycans or α-Dg core protein during the differentiation process in Kasumi-1 cells, we performed flow cytometry analysis using specific antibodies. Diminished expression not statistically significant of laminin-binding glycans was detected by IIH6C4 antibody for differentiated Kasumi-1, whereas only a small variation of α-Dg core protein expressed was detected by 6C1 antibody (cranin). Consistently, a great decrease in the number of laminin-binding glycans was detected by VIA4 antibody in differentiated Kasumi-1 cells. The expression of β-Dg and β-DgPY892 was also diminished in differentiated Kasumi-1 cells (Fig 2C).

To corroborate the decreased level of expression of dystroglycan subunits in non-differentiated and differentiated states observed by WB, IF and FACS assays, we analysed messenger RNA (mRNA) expression of d-Kasumi-1 and nd-Kasumi-1 cells by quantitative reverse transcription PCR (RT-qPCR). Fig 2D, shows that mRNA expression of dystroglycans was significantly higher in non-differentiated Kasumi-1cells compared to differentiated Kasumi-1 cells (p<0.05).

### Hypoglycosylated α-dystroglycan maintains its laminin-1 binding properties in differentiated Kasumi-1 cells

**Fig 3:**
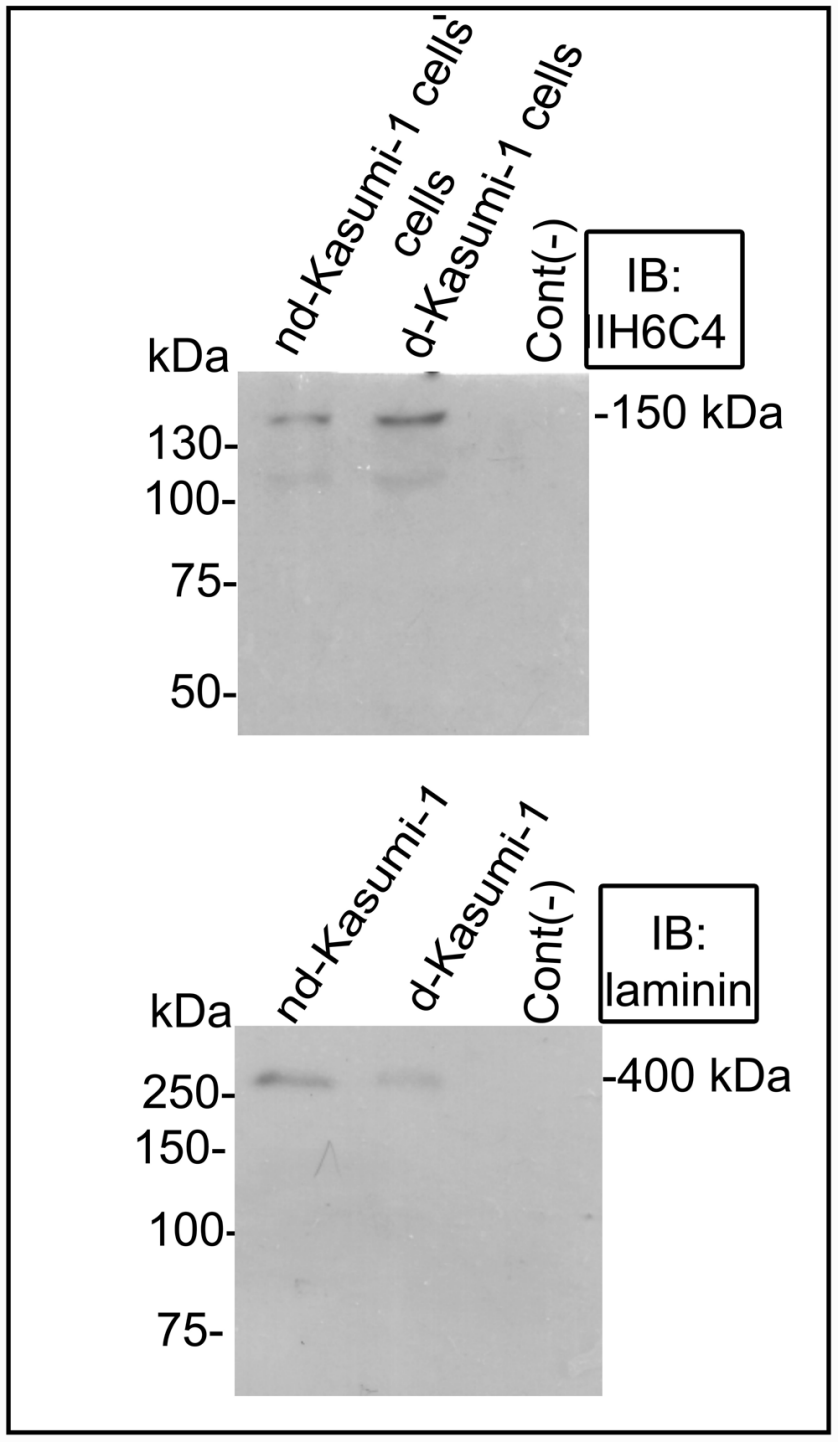
Laminin overlay assay. WGA-purified preparations from differentiated (d-Kasumi-1) and non-differentiated (nd-Kasumi-1 cells) compartments of Kasumi-1 cells were analysed by laminin overlay assay and Western blotting with IIH6C4 and laminin as a control.

To determine if the reduction of α-dystroglycan glycosylation detected by FACS and WB analysis in dKasumi-1 cells compared to ndKasumi-1 cells affected the ability of α-Dg to bind laminin, we performed a laminin overlay assay using cell extracts enriched by WGA affinity chromatography. WB with IIH6C4 antibody against the sugar chain moieties of α-DG showed the corresponding band of 150 kDa, while Laminin-1 was assayed as a control displaying a band of 400 kDa (Fig 3).

### Biochemical fractionation of dystroglycan

**Fig 4:**
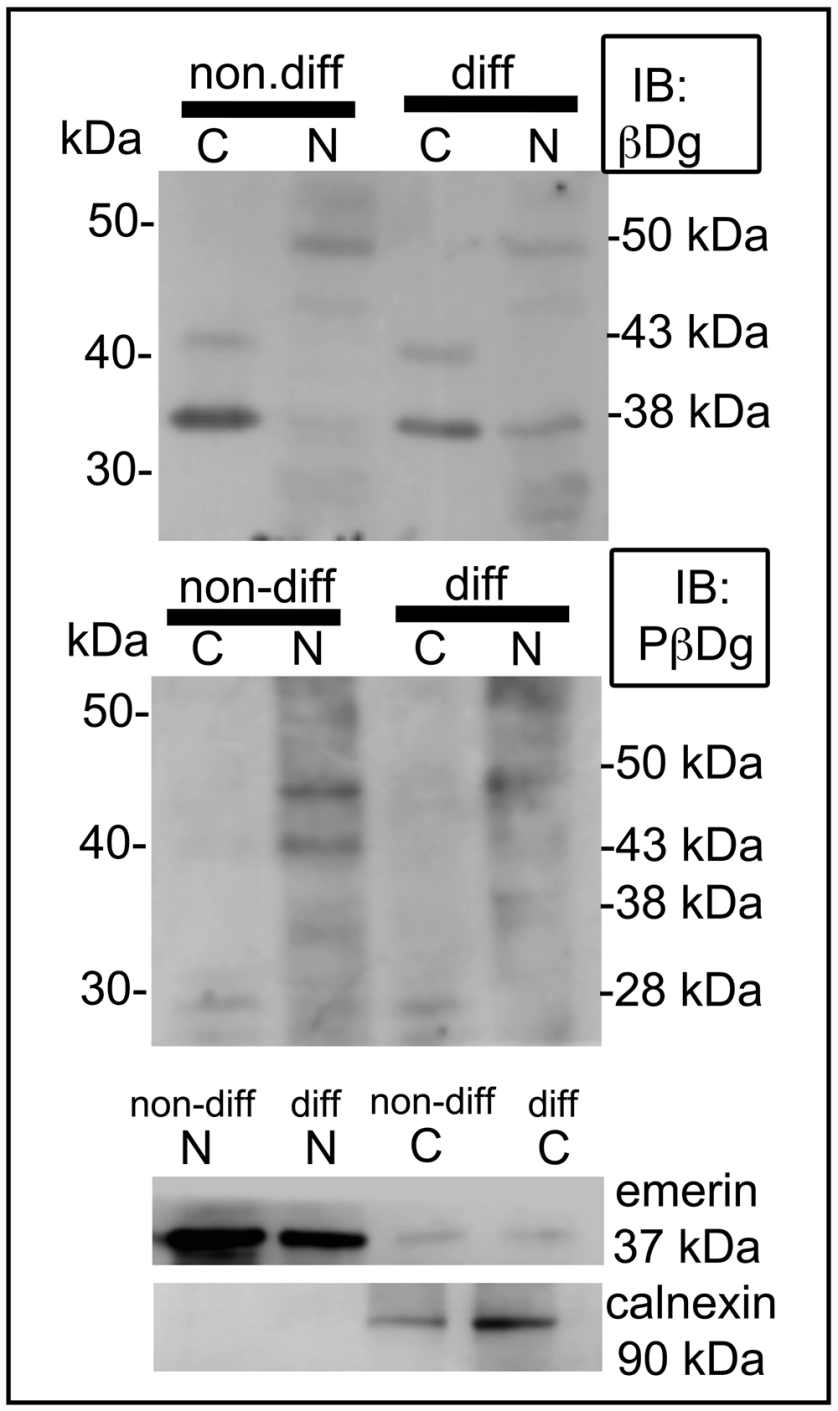
Cell fractionating and Western-blotting analysis of dystroglycan. Cellular fractionation of non-differentiated Kasumi-1 cells (nd-Kasumi-1 cells) and differentiated-Kasumi-1 cells (d-Kasumi-1 cells) revealed the presence of dystroglycan of 43 kDa and a prominent band of 38 kDa that were observed in the cytoplasm (C) fraction. This later band was also observed in a lesser extent at the nuclear (N) fraction (upper panel). For Tyrosine phosphorylated β-dystroglycan (lower panel) the predominant nuclear species was 43 kDa and 50 kDa for nd-Kasumi-1 cells and only a 50 kDa band was observed for dKassumi-1 cells. The 28 kDa fragment remained in the cytoplasmic (C) fraction of both kind of cells. Emerin and calnexin are shown as nuclear and cytoplasmic markers respectively.

To further investigate Dg species observed in different cell compartments of Kasumi-1 cells, we carried out biochemical fractionation of nucleus and cytoplasm of differentiated and non-differentiated Kasumi-1 cells (Fig 4).

Western blots performed at nuclei of non-differentiated Kasumi-1 and differentiated Kasumi-1 cells extracts revealed bands of 50 kDa, 38 kDa and 30 kDa nuclear dystroglycan, while the majority of full-length 43 kDa β-Dg was found mainly in the cytoplasmic fraction, the band of 38 kDa was also abundant in the cytoplasm of nd-Kasumi-1 and d-Kasumi-1 cells. The use of an antibody specific for β-dystroglycan phosphorylated on Y892, revealed bands of 50 kDa, 43 kDa, and 31 kDa, which were detected, in both cytoplasmic and nuclear fractions of non-differentiated Kasumi-1 and differentiated Kasumi-1 cells. The phosphorylated 31 kDa fragment of dystroglycan is the specie more translocated to the nucleus of non-differentiated cells while the 50 kDa was the more abundant specie at the nucleus of differentiated cells.

### Effect of silencing DG on dKasumi-1

**Fig 5:**
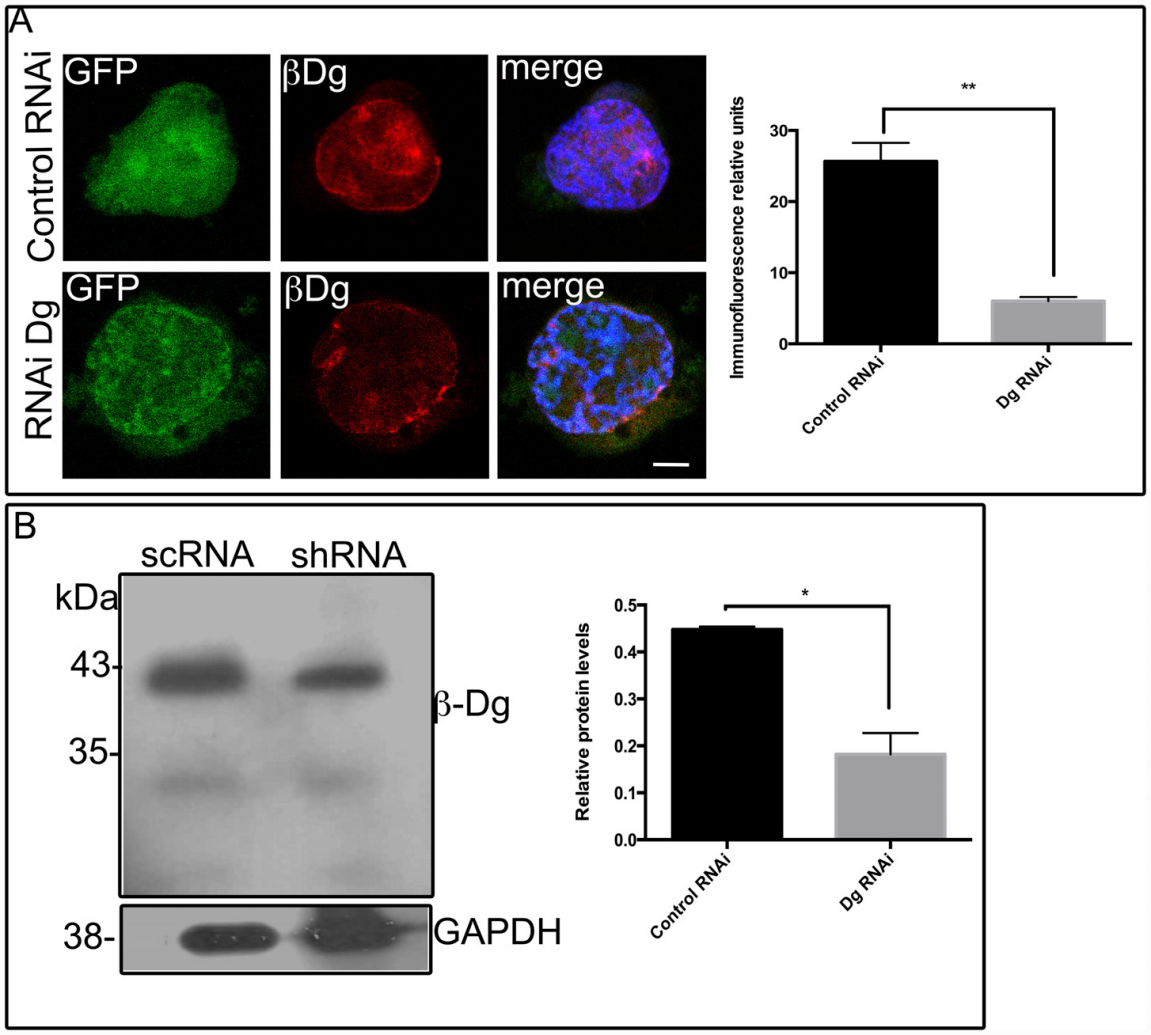
RNAi mediated depletion of β-Dystroglycan in Kasumi-1 cells. (A) Kasumi-1 cells expressing either control RNAi or Dg RNAi were immunolabeled for β-dystroglycan and analysed for confocal microscopy. Relative fluorescence units showed a significant depletion of β-Dg. Values shown are mean ±SD from three independent experiments (n = 3), respectively. **P<0.002. (B) Total Kasumi-1 cells extracts expressing either control RNAi or Dg RNAi were processed for Western-blot, utilizing an antibody against β-dystroglycan, corresponding bands were observed at 43 kDa. Densitometry analysis demonstrated β-Dg knockdown (51%) in cells transfected with a Dg iRNA as compared with cells transfected with a control iRNA. Values shown are mean ±SD from three independent experiments (n = 3), respectively. * P<0.05.

Differentiation of Kasumi-1 cells resulted in a decrease in dystroglycan expression (Fig 1). It was therefore of interest to ascertain whether β-Dg is involved in the physiology of promyelocytic leukaemia cells. In order to test this we analysed how β-Dg depletion influences the differentiation process of Kasumi-1 cells by employing a small interfering RNA (RNAi) to target dystroglycan (Dg RNAi). The RNAi was co-expressed with GFP to identify unequivocally individual RNAi-treated cells. As negative control, RNAi that is predicted not to block the translation of any specific gene was utilized (control RNAi) [9]. The effectiveness of RNAi treatment in reducing β-dystroglycan expression in these cells was evaluated by WB and IF assays. Global immunolabeling of β-Dg was reduced by more than 50% in GFP-expressing cells that co-express Dg-specific RNAi, compared with control RNAi-treated cells (Fig 5A). These levels of knockdown are in keeping with studies in other cell lines using the same targeting sequences [9]. Intensity of the β-dystroglycan immunoreactive band (~43 kDa) was decreased by ~51% in cell lysates of Kasumi-1 cells transiently transfected with vector expressing Dg RNAi but not in those expressing the control RNAi (Fig 5B). In parallel with normal macrophages, TPA-differentiated Kasumi-1 cells exhibit vacuole, nuclear lobulation, expression of the cell surface antigen CD68, and phagocytic capability [7] as we confirmed.

**Fig 6:**
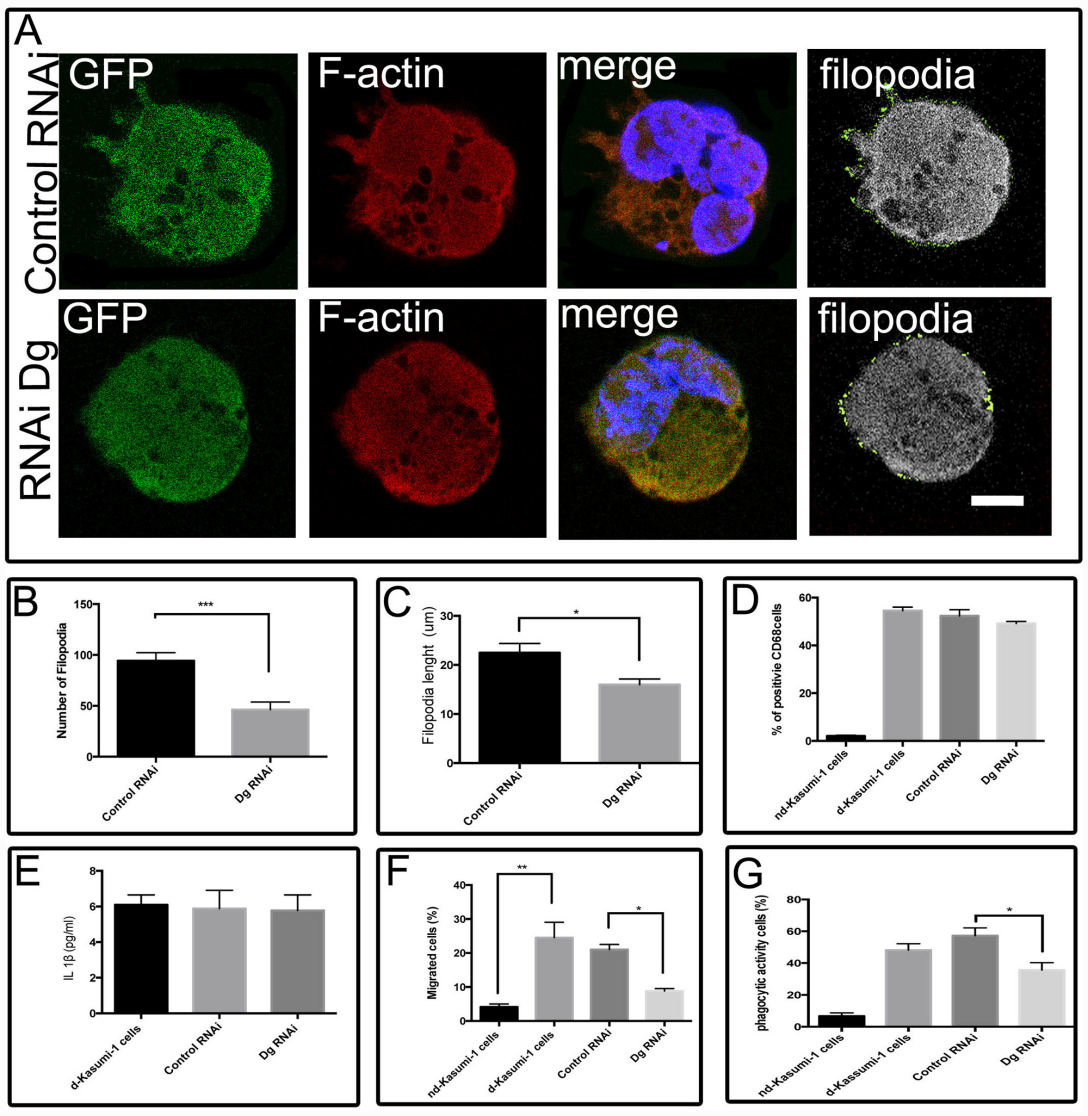
β-Dystroglycan-depleted Kasumi-1 cells impairs actin-based structures. (A) Kasumi-1 cells transfected with control and interference vector and differentiated were analysed by confocal microscopy to evaluate morphological changes in response to knockdown of dystroglycan. (B) The number of filopodia was quantified by the FiloDetect software in 20 Kasumi-1 RNAi-transfected cells that showed a low number of filopodia in relation to 20 control cells. Values shown are mean ±SD from three independent experiments (n = 3), respectively. *** P<0.005. (C) The length of filopodia was quantified by the FiloDetect software in 20 Kasumi-1 RNAi-transfected cells that showed shorter filopodia in relation to 20 control cells. Values shown are mean ±SD from three independent experiments (n = 3), respectively. * P<0.05. (D) CD68 levels were determined by flow cytometry. Relative CD68 levels obtained in control RNAi were set at 100%. Values shown are mean ±SD from three independent experiments (n = 3), respectively. (E) The secretion of IL-1β was measured and quantified by an ELISA kit of supernatants from control RNAi- or Dg RNAi-treated Kasumi-1 cells. Values shown are mean ±SD from three independent experiments (n = 3), respectively. (F) The efficiency of migration was measured with a Transwell chamber and Dg-depleted Kasumi-1 cells were less motile than control cells. Values shown are mean ± SD from three independent experiments (n = 3), respectively. ***P<0.005. (G) Phagocytic ability is lost in Dg RNAi treated Kasumi-1 cells compared to control Kasumi-1 and non-treated dKasumi-1 cells. Error bars show ±SD, based on a total of three experiments; * = p<0.05. nd-Kasumi-1 cells:- non-differentiated Kasumi-1 cells; d-Kasumi-1 cells.- differentiated Kasumi-1 cells.

To ascertain whether β-dystroglycan depletion affects Kasumi-1 cells, control and β-dystroglycan-depleted Kasumi-1 cells (50 X10^3^ cells) were processed for double immunostaining to detect the presence of morphological changes following differentiation (Fig 6A). The presence of filopodia was visualized with Filopodia_Code software (Fig 6A, right side, shown in green). β-Dg-depleted differentiated cells exhibited a significant diminution in the number of filopodia formed (mean 47 per cell) compared to control differentiated cells that protruded a mean of 94 per cell (Fig 6B), whilst filopodia length was reduced from 20 μm to 15 μm respectively (Fig 6C).

To further confirm if Dg expression affects the differentiation process in Kasumi-1 cells we monitored in 50 X 10^3^ cells, the expression of CD68, a well-established surface marker for differentiation. There was no difference in CD68 expression in β-dystroglycan-depleted dKasumi-1 cells compared to control dKasumi-1 cells (Fig 6D). To evaluate if Dg down-regulation affected the ability of cells to produce IL-1β a property related to the activation process, we analysed by ELISA the secretion of IL-1β of 50 X 10^3^ cells after treatment with LPS. Our results did not show any difference between the capabilities of macrophage-like cells with Dg depleted compared to differentiated non-transfected cells (Fig 6E). To test the functional significance of the changes in number and length of filopodia we performed migration assays in Transwell chamber of 50 X 10^3^ cells. Non-transfected differentiated and non-differentiated Kasumi-1 cells were included as controls. Dg depletion significantly reduced the migration of differentiated Kasumi-1 cells compared to cells treated with RNA control. Dg RNAi halved migration of differentiated Kasumi-1 cells to LPS in relation to the RNA control (Fig 6F). The phagocytic activity was present in 35.6% of Dg down-regulated differentiated Kasumi-1 cells compared to 57.2% of Dg control in differentiated Kasumi-1 cells. Additional controls of non-differentiated and differentiated non-transfected Kasumi-1 cells showed 6.6% and 48% of phagocytic activity respectively (Fig 6G).

**Fig 7:**
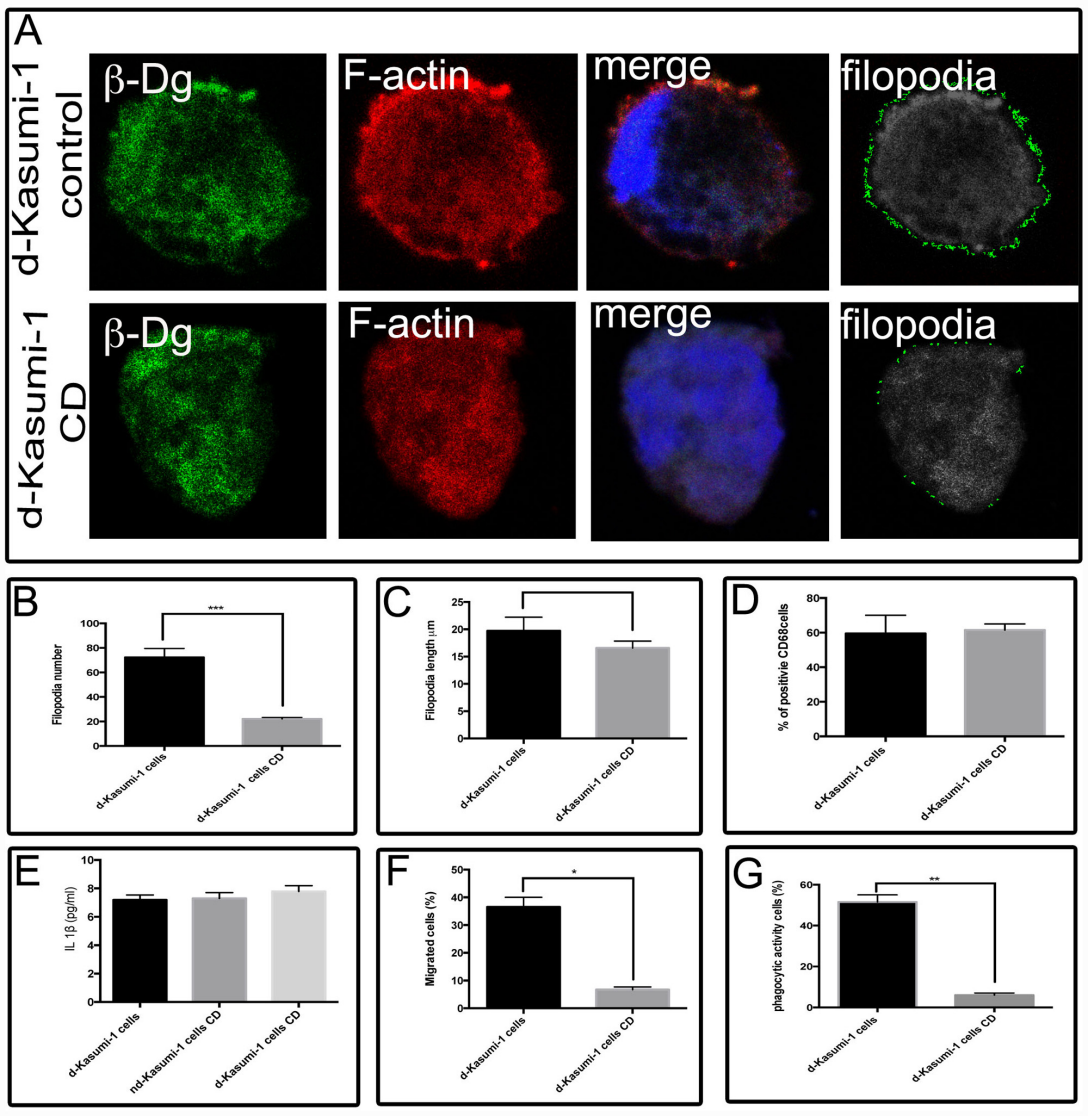
Cytochalasin D interferes with actin- dependent functions. (A) Differentiated (d-Kasumi-1 cells) and non-differentiated Kasumi-1 cells (nd-Kasumi-1 cells) treated with cytochalasin D (CD) were analysed by confocal microscopy to evaluate morphological changes in response to actin depolimerization. (B) The number of filopodia was quantified by the FiloDetect software in 20 Kasumi-1 CD treated cells that showed a low number of filopodia in relation to 20 control cells. Values shown are mean ±SD from three independent experiments (n = 3), respectively. *** P<0.005. (C) The length of filopodia was quantified by the FiloDetect software in 20 Kasumi-1 CD treated cells that showed shorter filopodia in relation to 20 control cells. Values shown are mean ±SD from three independent experiments (n = 3), respectively. * P<0.05. (D) CD68 levels were determined by flow cytometry. Relative CD68 levels obtained in control Kasumi-1 cells were set at 100%. Values shown are mean ±SD from three independent experiments (n = 3), respectively. (E) The secretion of IL-1β was measured and quantified by an ELISA kit of supernatants from control and CD-treated Kasumi-1 cells. Values shown are mean ±SD from three independent experiments (n = 3), respectively. (F) The efficiency of migration was measured with a Transwell chamber and CD treated Kasumi-1 cells were less motile than control cells. Values shown are mean ± SD from three independent experiments (n = 3), respectively. ***P<0.005. (G) Phagocytic ability is almost lost in CD treated Kasumi-1 cells compared to control Kasumi-1. Error bars show ±SD, based on a total of three experiments; * = p<0.05. d-Kasumi-1.-differentiated Kasumi-1 cells; CD.- Cytochalasin D.

To confirm that β-dg actively participates in actin-based structures and functions we processed differentiated Kasumi-1 cells treated with cytochalasin D (CD) an inhibitor of actin polimerization. Actin-dependent functions (number and length of filopodia, migration and phagocytic activity) were affected by actin disorganisation. Number and length of filopidia diminished with a mean of 72 and 36.5 respectively compared to controls 22 and 14.65 (mean value) respectively. Cytochalasin D treated cells showed a mean of 7 migrated cells and phagocytosis capability was also diminished to a mean value of 6 cells, compared to controls 67 and 51 respectively. Actin-independent functions (CD68 expression and IL-1β secretion) remained unaffected. Results of these experiments are shown in Fig 7.

## Discussion

We recently demonstrated the subcellular distribution and pattern of expression of Dg in a promyelocytic cell line HL-60, suggesting the active participation of Dg in granulocytic differentiation [9]. We were interested therefore to investigate whether Dg might have a similar key role in macrophage-like differentiation. In the present study we characterised the pattern expression and subcellular distribution of Dg in a human myeloid leukaemia cell line (Kasumi-1) [7], our results are indicative of Dg role as a scaffold in actin-based structures.

The molecular mass of α-Dg varies from 120 to 180 kDa, depending on the extensive glycoslylation, [13]. Using VIA4 and cranin antibodies that poorly recognise higher molecular weight α-DG species [14], we determined the expression of a prominent band of 80 kDa as well as bands of 70 kDa and 60 kDa in non-differentiated conditions; but not in differentiated Kasumi-1 cell extracts (Fig 1). These species might correspond to the molecular mass of the hypoglycosylated protein or could correspond to degradation products; although Kasumi-1 cells were cultured in the presence of an inhibitor of proprotein convertase (PC), which expression in human cancer cells increases both proliferation and invasive ability [15]. The presence of lower molecular mass α-Dg species might also suggest a failure in the posttranslational processing of dystroglycan, rather than in the synthesis of α-dystroglycan itself. Therefore we can not discard the presence of mutations in one of the seven glycosyltransferases or glycosyltransferase-like genes, that have been found to be involved in α-Dg functional glycosylation [6]. Enrichment of cell extracts with WGA and the use of IIH6C4 antibody let us determine the presence of the full-glycosylated α-Dg 150 kDa band.

Our flow cytometry analysis showed a reduced detection of α-Dg, based on its loss of reactivity to antibodies that recognize the laminin binding glycoepitope of α-Dg (IIH6C4 and VIA4-1) specifically in dKasumi-1 cells. This diminution was less evident for the reactivity observed with IIH6C4 and absent for the α-Dg core protein (cranin antibody).

Therefore, the diminutions detected are attributable to a specific reduction of laminin-binding glycan formation on α-Dg [16] and are inherent to the differentiation process. This hypoglycosylation is likely to reduce the functionality of α-Dg as a receptor for its known extracellular matrix proteins, including laminin, neurexin, and agrin [17]. However, our overlay assays, showed a residual laminin-binding detected from α-Dg 150 kDa specie. It has been reported that laminin-1 binding pattern is retained after enzymatic deglycosylation [18], this might suggest that Kasumi-1 cells might have additional laminin binding epitopes, which are less susceptible to deglycosylations. It is important to consider the existence of integrins, that have been reported to function as laminin receptors [19].

*In vitro* differentiated macrophages and myelomonocytic cell lines showed down-regulation of adhesion molecules after TPA treatment [20]. We hypothesised that under physiological conditions, during macrophage-like generation cells Dg is also downregulated, facilitating cell recruitment to solid tissues.

The presence of a nuclear localisation sequence (NLS) has been described in β-Dg [21], therefore the presence of β-Dg and specially β-DgPY892 in the nucleus of dKasumi-1 cells might indicate its potential role as a scaffold during the differentiation process, which is characterised for the change of nuclear shape from rounded to lobular. The role of dystroglycan as an organiser of nuclear architecture has been documented; as well as the interaction of β-Dg with other nuclear proteins including emerin and lamin B1 that could contribute to a role for modulation of transcriptionally active regions in the nucleus [22,23].

Recently, it has been described that full-length β-Dg (43 kDa) was the predominant specie found in the nuclear fraction, whereas for phosphorylated β-Dg the 31 kDa transmembrane and cytoplasmic fragment was more prominent in the nucleus. Our cell fractioning assays, showed that the 43 and 38 kDa β-Dg proteins were mainly located in the cytoplasm fraction, while a 50 kDa specie was observed in the nuclear fraction as has been reported for prostate tissues [23]. According to our results, Dg species distribution in different cell compartments should play a particular role depending on its physiological stage, the tissue or even on the cell lineage if we compare with HL-60 cells (promyelocytic leukaemia cell line) [9].

The Kasumi-1 cell line is a model system of Acute Myeloid Leukaemia with t(8;21) translocation, and the corresponding functional consequences of the AML1-ETO fusion oncogene on myeloid differentiation [24]. Nevertheless leukemogenesis involves multiple genetic changes that impair hematopoietic differentiation, it is believed that signalling by tyrosine kinases other than KIT may be responsible for proliferative and/or survival advantage in Kasumi-1 cells [25]. It could be therefore, that the activation of other tyrosine kinases during the differentiation process, such as Src [26,27], could lead to the increased levels of phosphorylated dystroglycan observed.

The knockdown of Dg slows dKasumi-1 cells movement as well as the maintenance of a more rounded shape and altered filopodia extension compared to control cells (Fig 6). Similar phenotypes have also been described for oligodendrocytes and HL-60 cells [9,28]; in addition, alteration in filopodia number and length also affect concomitant cell motility and phagocytic capabilities. Dystroglycan has been proposed to be important for the maturation of adhesions into fibrillar adhesions and therefore in long-term adhesion and migration cells [29]. Our results demonstrate that a direct consequence of the reduction in dystroglycan have an effect on actin cytoskeletal dynamics, through its binding to actin, and also modulating the actin regulatory machinery through RhoGDI [4,30,31].

Treatment of cells with cytochalasin D demonstrated that the functions affected by dystroglycan depletion depend on actin cytoskeleton such as phagocytosis, migration, number and length of filopodia, according to the experiments showed in Fig 7.

Kasumi-1 cells as an in vitro biological model mimic the acute myeloblastic leukaemia and although they only recapitulate parts of the disease, they are critical to discern mechanisms of normal differentiation process and they help to explain the leukaemia progression in which dystroglycans play an important role.

## Conclusion

Our findings strongly suggest the participation of dystroglycan as a scaffolding protein that is part of actin-based structures, and actively participates in filopodia extrusion, phagocytosis, and migration that were evident in Kasumi-1 cells differentiated to macrophage-like cells.

## Supporting Information

S1 Fig Specific contribution of phosphorylated dystroglycan in Kasumi-1 cells. (A) Percentage of the specific contribution of β-DgPY892 (31 kDa band) in relation to the rest of the proteins detected with the respective antibody in nd-Kasumi-1 cells. B. Percentage of the Specific contribution of β-DgPY892 (31 kDa band) in relation to the rest of the proteins detected with the respective antibody in dKasumi-1 cells.
(EPS)
